# Genome-wide identification and characterization of genes involved in carotenoid metabolic in three stages of grapevine fruit development

**DOI:** 10.1038/s41598-017-04004-0

**Published:** 2017-06-26

**Authors:** Xiangpeng Leng, Peipei Wang, Chen Wang, Xudong Zhu, Xiaopeng Li, Hongyan Li, Qian Mu, Ao Li, Zhongjie Liu, Jinggui Fang

**Affiliations:** 1 College of Horticulture, Nanjing Agricultural University, Tongwei Road 6, Nanjing, 210095 P.R. China; 2 Grape and Wine Research Institute, Guangxi Academy of Agricultural Sciences, Daxuedong Road 174, Nanning, 530007 P.R. China; 3 Shandong Aacademy of Grape, Gongyenan Road 103, Jinan, 250110 P.R. China

## Abstract

Carotenoids not only play indispensable roles in plant growth and development but also enhance nutritional value and health benefits for humans. In this study, total carotenoids progressively decreased during fruit ripening. Fifty-four genes involving in mevalonate (MVA), 2-C-methyl-D-erythritol 4-phosphate (MEP), carotenoid biosynthesis and catabolism pathway were identified. The expression levels of most of the carotenoid metabolism related genes kept changing during fruit ripening generating a metabolic flux toward carotenoid synthesis. Down regulation of *VvDXS*, *VvDXR*, *VvGGPPS* and *VvPSY* and a dramatic increase in the transcription levels of *VvCCD* might be responsible for the reduction of carotenoids content. The visible correlation between carotenoid content and gene expression profiles suggested that transcriptional regulation of carotenoid biosynthesis pathway genes is a key mechanism of carotenoid accumulation. In addition, the decline of carotenoids was also accompanied with the reduction of chlorophyll content. The reduction of chlorophyll content might be due to the obstruction in chlorophyll synthesis and acceleration of chlorophyll degradation. These results will be helpful for better understanding of carotenoid biosynthesis in grapevine fruit and contribute to the development of conventional and transgenic grapevine cultivars for further enrichment of carotenoid content.

## Introduction

The isoprenoids, also known as terpenoids and terpenes, are the largest class of plant secondary metabolites and have numerous biochemical functions in plants. They play pivotal role as photosynthetic pigments (e.g., carotenoids and phytol), plant hormones (e.g., gibberellins, strigolactones and brassinosteroids), electron carriers (e.g., plastoquinone), and as plant defense compounds as well as attractants for pollinators (monoterpenes, sesquiterpenes, and diterpenes)^1, 2^. Carotenoids are a sungroup of isoprenoid molecules with more than 750 members occurring throughout the natural world and participate in various physiological and developmental processes in plants^3, 4^. In photosynthetic green tissues, carotenoids play an essential role in photosynthesis for photosystem assembly, light harvesting, and photoprotection^5^. In non-photosynthetic tissues, carotenoids provide bright colors and produce aromas and flavors to attract insects and animals for pollination and seed dispersal^6^. Carotenoids also serve as precursors for two important phytohormones, abscisic acids (ABA) and strigolactones, which are key regulators for plant development and stress response^7–9^. In addition, increasing interest is devoted to carotenoid content and composition of food crops because of their important roles in human nutrition and health^10, 11^.

Like all isoprenoids, carotenoids are synthesized from the five carbon units isopentenyl diphosphate (IPP) and its double-bond isomer dimethylallyl diphosphate (DMAPP)^12, 13^. Two pathways exist in plant cells for the production of these prenyl diphosphate precursors, but carotenoids are mainly synthesized from IPP and DMAPP produced by MEP pathway, as shown in Supplementary Fig. S1. The first committed step of carotenoid biosynthesis is the production of 40-carbon phytoene from condensation of two GGPP molecules (Fig. S1). This reaction, catalyzed by the enzyme phytoene synthase (PSY), is considered the main bottleneck in the carotenoid pathway^12^.Phytoene is then desaturated and isomerized to all-trans-lycopene through the action of two desaturases and two isomerases: phytoene desaturase (PDS), ζ-carotene desaturase (ZDS), prolycopene isomerase (CRTISO) and ζ-carotene isomerase (ZISO). The formation of δ-carotene and γ-carotene from lycopene are catalyzed by lycopene ε-cyclase (LCYE) and β-cyclase (LCYB), and then the orange α-carotene and β-carotene are synthetized by LCYB. Finally, these carotenes are transformed into lutein and zeaxanthin by heme and non-heme β-carotene hydroxylases (CYP97 and CHYB). Zeaxanthin is converted to violaxanthin by the action of zeaxanthin epoxidase (ZEP) and further to neoxanthin by the action of the neoxanthin synthase (NXS). These two xanthophylls are cleaved by 9-cis-epoxycarotenoid dioxygenase (NCED), a key enzyme in the biosynthesis of ABA^4–6, 14–16^.

Grapevine (*Vitis*) is one of the most commonly consumed and widely cultivated fruit crop worldwide^17–19^. Due to its important nutritional values and health benefits, grapevine becomes the most popular and important in the diets of people throughout the world. The carotenoid content and composition of grape berry has received considerable attention due to their potential precursors to a group of potent aroma compounds (C13-norisoprenoids) in grapevines and wines^17^. To maximize the health-promoting benefits of carotenoids through increased consumption, characterization of carotenoid synthesis and accumulation in important food crops such as grapevine is essential. A complete understanding of the carotenogenesis genes is fundamental for elucidating the mechanisms of carotenoid biosynthesis in grapevine, as well as for the breeding of new grapevine varieties with rich carotenoids, which are good for human health. With the release of the grapevine genome sequence and with the increasing affordability of high throughput analysis tools^20, 21^, there will be a better opportunity to systematically study the carotenogenesis genes in grapevine. In this study, 54 carotenoid biosynthetic genes were identified in grapevine genomic. Transcriptome were used to profile the expression of carotenoid biosynthetic/catabolic genes during grape berry development and ripening. Carotenoid concentrations were also determined at three distinct stages of berry development: green, véraison and ripe/harvest stages. The systematic analysis of carotenoid biosynthesis genes in grapevine will improve our understanding of the genetic mechanisms of carotenoid biosynthesis and carotenoid accumulation in grapevine.

## Results

### Identification of 54 genes involving in grapevine carotenoid biosynthetic and catabolic pathway

To determine the molecular basis and mechanism of the carotenoid biosynthetic/catabolic during grapevine fruit development, the genes involving in grapevine carotenoid biosynthetic/catabolic pathway were identified from the current genome. A total of fifty-four carotenoid biosynthesis-related genes were identified via BLAST-P search in NCBI using the Arabidopsis gene sequences as queries^1, 15^. Subsequently, to verify the reliability of the initial results, a survey was conducted to confirm these genes using functional annotation of the grapevine transcriptome in three distinct stages of berry development (NCBI GEO Accession: GSE77218)^19^. As a result, 54 non-redundant carotenoid biosynthesis-related genes were identified and each carotenoid biosynthetic gene in grapevine was named based on the enzymatic reaction, similar to those given in the A. thaliana carotenoid biosynthetic pathway. Detailed information about each carotenoid biosynthetic and catabolic gene was showed in Table 1, including protein length, isoelectric points (pI), molecular weights, aliphatic index, grand average of hydropathicity (GRAVY) and subcellular localizations (Table 1).

**Table 1:** Carotenoid metabolic genes in grapevine.

Name	Accession no.	Protein Len	Chrom	Chr srart	Chr end	Mol.wt (kDa)	pI	Aliphatic index	GRAVY	Loc^a^
**MVA pathway**										
VvAACT1	VIT_00s0531g00050.t01	440	chrUn	31408786	31413407	45958.9	8.54	99.59	0.187	C
VvAACT2	VIT_12s0057g01200.t01	415	chr12	9967097	9973038	42339.6	5.82	100.19	0.261	—
VvHMGS	VIT_02s0025g04580.t01	464	chr2	4131692	4138165	51004.0	5.92	76.70	−0.192	—
VvHMGR1	VIT_18s0122g00610.t01	593	chr18	494573	498105	63636.3	6.83	92.45	0.072	M
VvHMGR2	VIT_04s0044g01740.t01	561	chr4	23597424	23599575	59777.0	7.86	94.39	0.175	—
VvHMGR3	VIT_03s0038g04100.t01	575	chr3	2970538	2972585	61190.6	6.07	97.36	0.160	—
VvMVK	VIT_14s0128g00330.t01	388	chr14	2969463	2974536	41209.6	5.96	102.81	0.259	S
VvpMVK	VIT_02s0012g02530.t01	508	chr2	10033966	10045076	55167.9	5.67	91.04	−0.100	—
VvMDC	VIT_13s0106g00790.t01	422	chr13	10644213	10652294	46640.1	6.48	85.07	−0.300	—
VvFPS	VIT_19s0015g01010.t01	341	chr19	9094526	9098457	39064.9	5.53	95.43	−0.201	—
**MEP pathway**										
VvDXS1	VIT_05s0020g02130.t01	716	chr5	3851283	3856072	77091.0	6.36	88.04	−0.078	C
VvDXS2	VIT_11s0052g01730.t01	735	chr11	19555292	19558837	78975.0	7.13	86.83	−0.133	C
VvDXS3	VIT_04s0008g04970.t01	719	chr4	4457279	4468718	78929.2	6.53	88.71	−0.018	C
VvDXS4	VIT_11s0052g01780.t01	690	chr11	19599532	19602913	74142.5	6.32	88.10	−0.119	C
VvDXS5	VIT_00s0218g00110.t01	718	chrUn	14030385	14035390	77179.6	7.92	88.87	−0.106	C
VvDXS6	VIT_11s0052g01240.t01	731	chr11	18968097	18972989	78991.5	7.22	90.26	−0.121	C
VvDXR	VIT_17s0000g08390.t01	471	chr17	9583558	9590206	51176.1	6.03	99.64	−0.016	C
VvMCT	VIT_12s0035g01950.t01	308	chr12	22263637	22269775	33830.0	6.54	100.88	−0.095	C
VvCMK	VIT_06s0009g02320.t01	394	chr6	14652972	14664719	43473.3	8.05	81.22	−0.270	C
VvMDS	VIT_02s0025g00380.t01	168	chr2	486537	487271	17970.5	6.74	91.85	−0.008	C
VvHDS	VIT_06s0004g02900.t01	740	chr6	3629087	3636025	82345.3	5.91	90.49	−0.255	C
VvHDR	VIT_03s0063g02030.t01	465	chr3	5305933	5311191	52522.5	5.32	80.88	−0.399	C
VvIDI	VIT_04s0023g00600.t01	293	chr4	16802127	16809421	33557.4	6.16	87.54	−0.318	C
**Carotenoid biosynthetic pathway**										
VvGGPPS1	VIT_04s0023g01210.t01	368	chr4	17612949	17614055	39801.9	5.91	97.80	−0.049	C
VvGGPPS2	VIT_18s0001g12 000.t01	371	chr18	10227177	10228292	39683.4	5.78	92.59	−0.058	M
VvGGPPS-LS	VIT_05s0020g01240.t01	276	chr5	2987258	2988130	29744.2	4.93	101.45	0.039	—
VvGGPPS-SS	VIT_19s0090g00530.t01	298	chr19	6656884	6657780	32785.3	6.19	84.87	−0.307	C
VvPSY1	VIT_04s0079g00680.t01	437	chr4	11495290	11499126	49643.7	8.42	82.61	−0.330	—
VvPSY2	VIT_12s0028g00960.t01	396	chr12	1467727	1470354	45152.8	9.15	84.72	−0.328	C
VvPSY3	VIT_06s0004g00820.t01	357	chr6	935500	940685	41210.1	6.25	82.02	−0.331	—
VvPDS	VIT_09s0002g00100.t01	582	chr9	70786	92779	65495.5	6.58	92.97	−0.183	M
VvZ-ISO	VIT_05s0062g01110.t01	365	chr5	19770533	19774697	40610.1	8.83	101.78	0.323	C
VvZDS	VIT_14s0030g01740.t01	552	chr14	6600703	6613497	61181.9	7.07	87.99	−0.178	—
VvCRTISO1	VIT_08s0032g00800.t01	641	chr8	4370241	4386309	70659.6	8.50	91.83	−0.054	—
VvCRTISO2	VIT_12s0035g01080.t01	570	chr12	20842885	20849118	62034.1	7.20	89.65	−0.094	C
VvLCYE	VIT_11s0016g01880.t01	529	chr11	1519660	1526200	59261.3	6.58	89.58	−0.064	C
VvLCYB	VIT_08s0007g05690.t01	508	chr8	19614960	19620336	57101.1	7.20	91.32	−0.094	—
VvCHYB1	VIT_02s0025g00240.t01	299	chr2	360270	361917	33057.2	8.40	90.03	0.032	C
VvCHYB2	VIT_16s0050g01090.t01	306	chr16	18017218	18018829	34217.5	9.58	80.95	−0.080	C
VvCYP97C	VIT_08s0007g04530.t01	546	chr8	18452646	18457877	61121.1	5.78	93.90	−0.107	C
VvCYP97A	VIT_04s0023g00080.t01	638	chr4	15934070	15957007	70813.5	6.56	92.92	−0.163	C
VvZEP1	VIT_07s0031g00620.t01	658	chr7	16796162	16803660	72087.5	7.93	83.43	−0.174	—
VvZEP2	VIT_13s0156g00350.t01	475	chr13	24068514	24072138	53119.2	8.74	91.52	−0.150	—
VvZEP3	VIT_00s0533g00020.t01	479	chrUn	31461160	31463786	52399.3	8.65	94.82	−0.103	C
VvVDE	VIT_04s0043g01010.t01	479	chr4	15682355	15687333	54596.4	6.03	77.29	−0.397	C
VvNXS	VIT_14s0006g02880.t01	246	chr14	21063839	21068184	27562.4	9.76	92.44	0.242	C
VvCCS	VIT_06s0080g00810.t01	497	chr6	20814283	20815776	56254.1	8.47	86.90	−0.167	C
**Carotenoid catabolism**										
VvCCD1	VIT_13s0064g00840.t01	546	chr13	22673076	22681735	61634.8	6.13	81.90	−0.271	—
VvCCD4a	VIT_02s0087g00910.t01	599	chr2	18560792	18562591	65900.3	6.88	82.35	−0.236	C
VvCCD4b	VIT_02s0087g00930.t01	589	chr2	18588968	18590737	65607.2	6.63	82.41	−0.187	C
VvCCD8	VIT_04s0008g03380.t01	546	chr4	2785955	2788636	60526.9	6.99	79.80	−0.324	C
VvNCED1	VIT_19s0093g00550.t01	609	chr19	17645484	17647313	67132.0	6.38	76.04	−0.317	C
VvNCED2	VIT_10s0003g03750.t01	605	chr10	6374536	6376353	67339.1	6.36	78.00	−0.365	C
VvNCED6	VIT_05s0051g00670.t01	575	chr5	11589343	11591070	63137.5	8.24	87.13	−0.202	C

^a^The subcellular location result of grapevine carotenoid metabolic genes by TargetP. ‘C’: Chloroplast; ‘M’: Mitochondrion; ‘S’: Secretory pathway; ‘—’ was any other location without chloroplast, mitochondrion, secretory pathway in cell.

Among the 54 carotenoid biosynthetic genes in grapevine, 10, 13, 24 and 7 genes were involved in MVA, MEP, carotenoid biosynthetic and catabolic pathways, respectively (Tables 1 and S1). These genes were members of 32 different gene families, and 22 genes were identified as single gene copy (Table 1). All these genes, only two (*VvPSY3* and *VvCCD8*) were not expressed with a very low number of reads sequenced and an extremely low RPKM (reads per kilo base of exon model per million reads) value during three ripening stages. One transcript of *VvMVK* showed no change in expression level (|log2 fold-change (log2FC)| < 0.25). 20 transcripts were observed as up-regulated or down-regulated slightly (0.25 < |log2FC| < 1). Thirty-one transcripts were considered as significantly differentially-expressed genes (|log2FC|) ≥ 1 (Table S1).

### Chromosomal distribution of grapevine biosynthetic genes

As shown in Fig. 1, 51 of 54 grapevine carotenoid biosynthetic genes were distributed unevenly throughout the 17 out of the 19 chromosomes, with eight genes located in Chromosome 4, while none of the carotenoid biosynthetic/catabolic genes mapped in Chromosomes 1 and 15 (Fig. 1). The remaining gene (*VvDXS5*, *VvAACT1* and *VvZEP3*) had not yet been assembled to any chromosome according to the current genome.

**Figrue 1:**
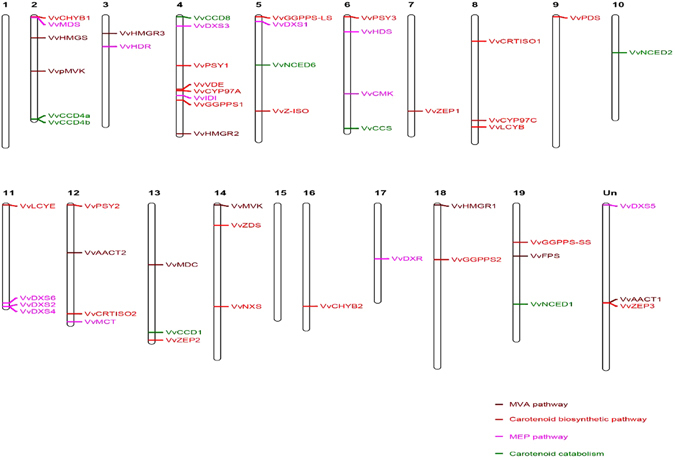
Chromosomal location of grapevine carotenoid metabolic genes.

Tandem and segmental duplications have been suggested to be two of the main causes for gene family expansion in plants^20^. The tandem duplication events were defined according to the methods of Holub^21^, where a chromosomal region within 200 kb containing two or more genes is defined as a tandem duplication event. There were five genes (VvDXS2/VvDXS4/VvDXS6 and VvCCD4a/VvCCD4b) clustered into two tandem duplication event regions on grape chromosome 2 and 11 (Table 1). Besides the tandem duplication events, 3 segregation duplication events (*VvHMGR1*/ *VvHMGR3*, *VvGGPPS1*/*VvGGPPS2* and *VvCHYB1*/*VvCHYB2*) were also identified (Fig. 2), indicating that some grapevine carotenoid biosynthetic genes were possibly generated by gene duplication. Moreover, the segregation duplication events can also provide a reference for the carotenoid biosynthetic gene evolutionary relationship and functional prediction.

**Figrue 2:**
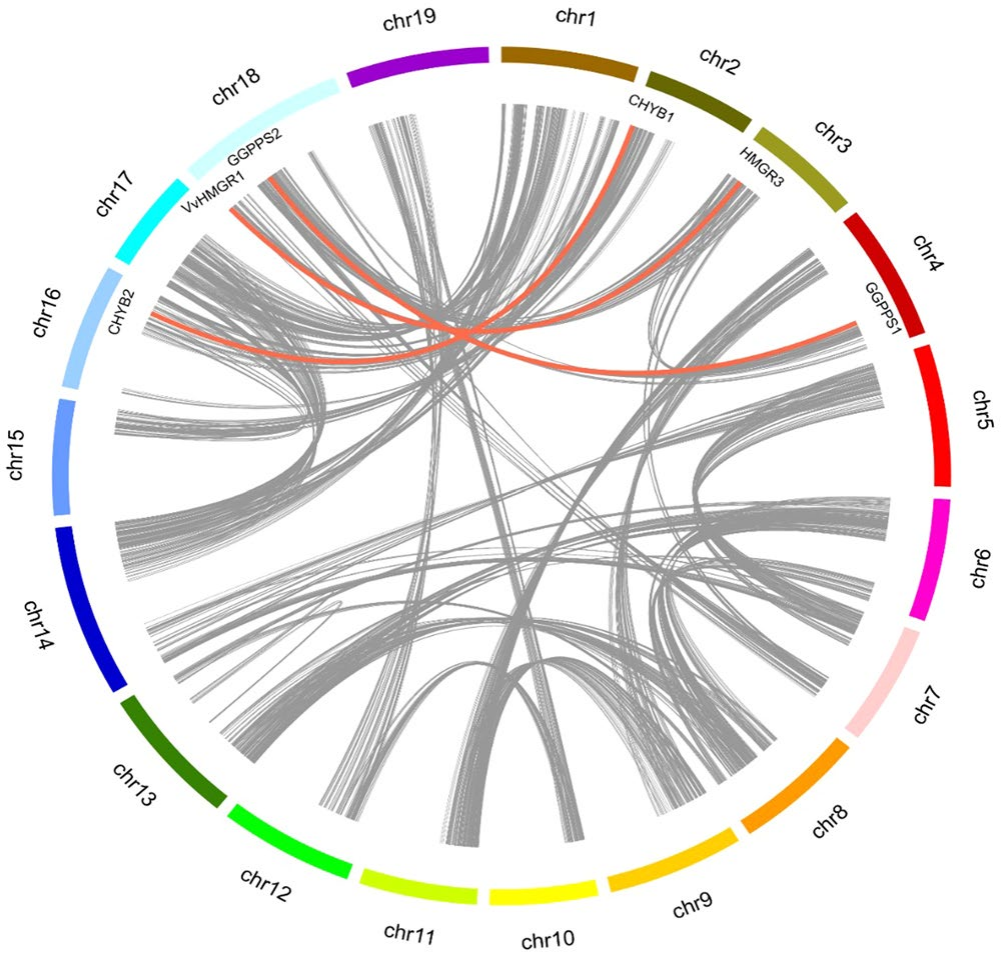
Synteny analysis of grapevine carotenoid metabolic genes. Gray lines indicate all synteny blocks in the grapevine genome, whereas the red lines suggest duplicated gene pairs.

### Exon-intron organization of grapevine carotenoid biosynthetic gene

The exon-intron structure analysis was performed to gain more insight into the grapevine carotenoid biosynthetic genes. High variation was observed in numbers of exons and introns among grapevine carotenoid biosynthetic genes (Fig. 3). Nine genes (*VvGGPPS1*, *VvGGPPS2*, *VvGGPPS-SS*, *VvCCS*, *VvCCD4a*, *VvCCD4b*, *VvNCED1*, *VvNCED2* and *VvNCED6*) had no intron in the grapevine genome. Most carotenoid metabolic genes contained 6 to 12 exons in their coding DNA sequences. However, *VvHDS*, the most exons contained in this study, had 19 exons in its coding DNA sequences. *VvCYP97A* was the longest carotenoid metabolic gene with 22 kb genomic sequence. The large variation in the structures of carotenoid metabolic genes indicated that the grapevine genomic had changed significantly during its extensive evolutionary processes.

**Figrue 3:**
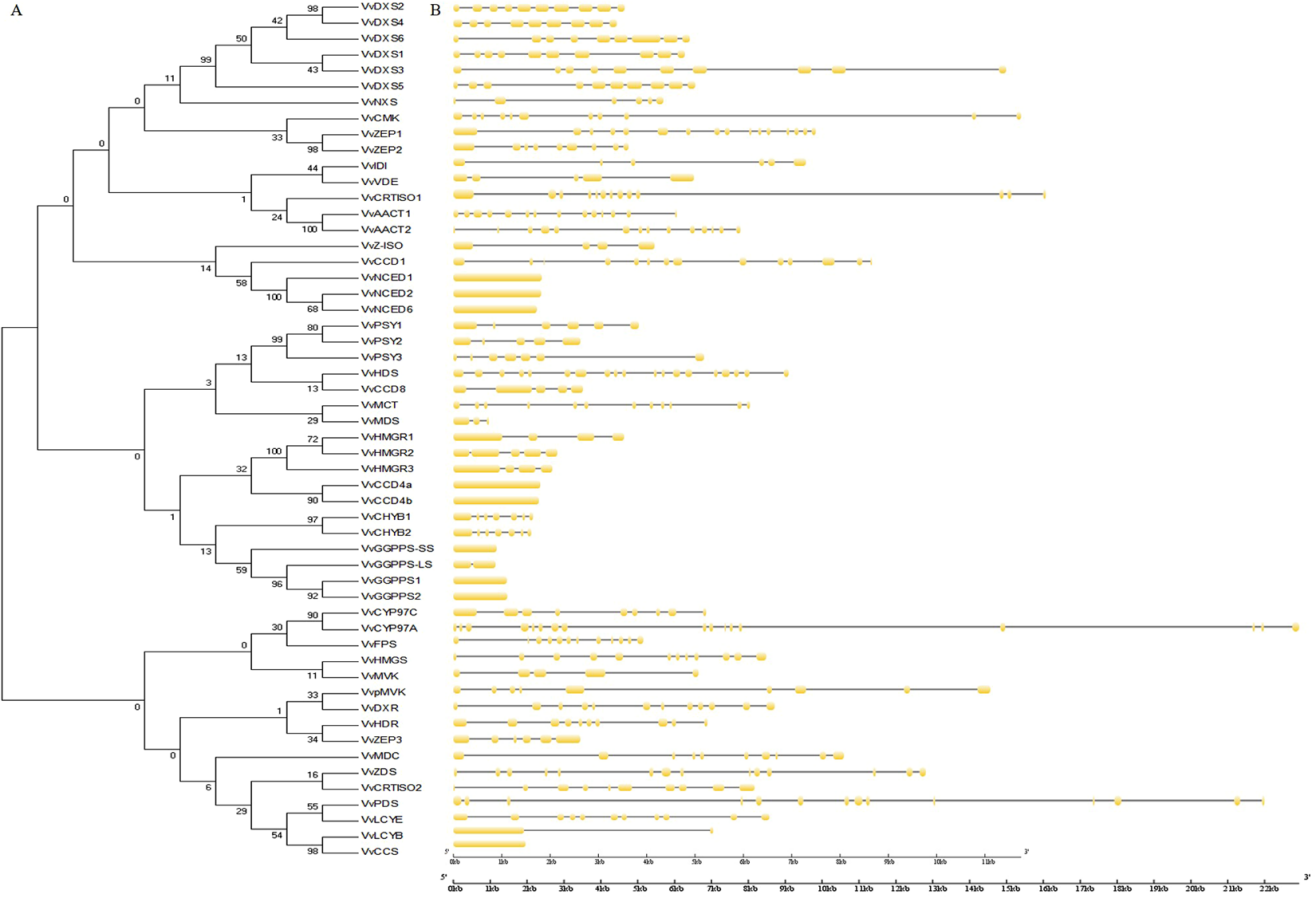
Structure analysis of carotenoid metabolic genes. (**A**) NJ phylogenetic tree of carotenoid metabolic proteins. (**B**) Exon-intron structure of carotenoid metabolic genes. Two different legends were used, 4 genes with long genomic sequence (*VvPDS*, *VvZDS*, *VvCRTISO1* and *VvCYP97A*) used the long legends, and the others genes used the short legends. Yellow indicates exons; black indicates introns.

### Variation of carotenoid and chlorophyll content during berry developing and ripening

In order to understand carotenoid and chlorophyll accumulation profiles in grapevine, the concentrations of carotenoid and chlorophyll in grape flesh were monitored at the three sampling time-points during fruit development and ripening (Fig. 4). It was observed that the amounts of carotenoid and chlorophyll decreased significantly throughout fruit ripening stages. As expected, the total amount of carotenoids (expressed as μg/g fw) progressively decreased from 7.51 μg/g fw at the green stage to 2.19 μg/g fw at the ripe stage (Fig. 4). Similarly, the total contents of chlorophyll a and chlorophyll b decreased 3.7 and 3.2-fold during ripening, respectively (Fig. 4). The ratios of carotenoids/chlorophylls (~0.18) as well as the ratio of chlorophyll a/b (~2.0) remained constant throughout the sampling stages. Our results were consistent with the previous finding that carotenoid and chlorophyll contents of grape berries decreased with ripening especially from veraison to harvest^22–24^.

**Figrue 4:**
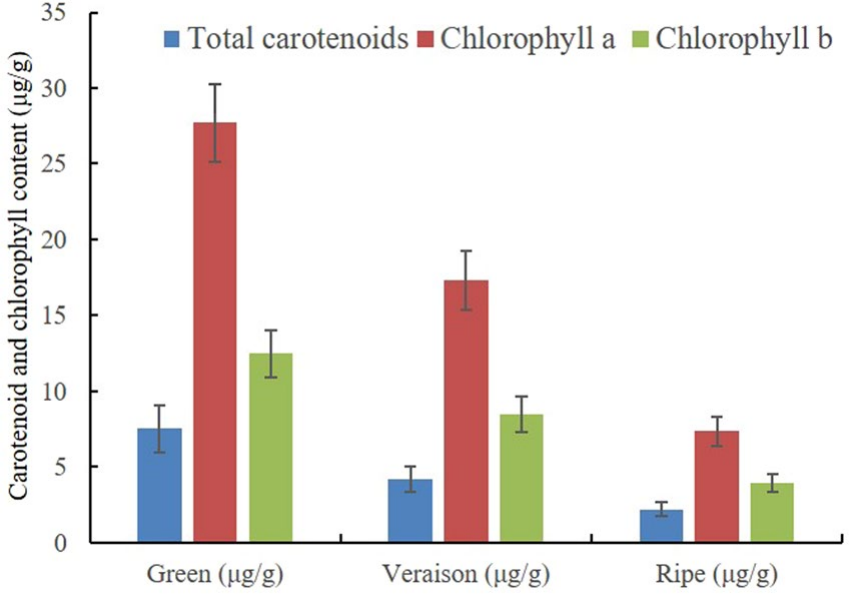
Total carotenoid and chlorophyll content during grapevine fruit ripening.

### Characterization and expression analysis of genes involving in the MVA pathway

Nine genes involved in six enzymatic steps were found in MVA pathway, which led to the formation of IPP and DMAPP. In addition, there was one *FPS* gene which catalyzed the synthesis of farnesyl diphosphate (FPP), the major substrate used by cytosolic and mitochondrial branches of the isoprenoid pathway^25, 26^.

Acetyl-CoA acetyltransferase (AACT) catalyses the condensation of two acetyl-CoA subunits to form acetoacetyl-CoA thus directing this central metabolite to the MVA pathway. In this study, *VvAACT1* and *VvAACT2* showed the highest expression levels at veraison and ripe stages, respectively. Interestingly, *Vv*ACAT2 mRNA expression (RPKM = 321.8 ± 71, the average value of the three ripening stages) had an approximate 5.8-fold higher than *VvAACT1* (RPKM = 55.0 ± 13, also the average value of the three ripening stages) during berry developmental stages, suggesting that this enzyme may divert the metabolic flux of acetyl-CoA from the biosynthesis of fatty acids and amino acids toward the synthesis of isoprenoids (Fig. 5 and Table S1). 3-Hydroxy-3-methylglutaryl-CoA reductase (HMGR), the first committed step of the MVA pathway for isoprenoid biosynthesis, catalyses the formation of MVA from 3-hydroxy-3-methyl-glutaryl-CoA (HMG-CoA)^27^. Three *HMGR* genes (*VvHMGR1*, *VvHMGR2* and *VvHMGR3*) were identified in grapevine and two members (*VvHMGR1* and *VvHMGR3*) were generated by gene duplication (Fig. 2). This was in agreement with previous reports that plant *HMGR* genes had arisen by gene duplication and subsequent sequence divergence^28^. Three members of *VvHMGR* exhibited significant differential expression patterns during fruit development. *VvHMGR1* and *VvHMGR2* showed the expression peak at green berry stage, whereas the highest expression of *VvHMGR3* was detected at ripe stage (Fig. 5 and Table S1), suggesting that *VvHMGR* might play a complicated role in the MVA pathway. Furthermore, the last three enzymes were encoded by single gene in grapevine. The expression of three genes exhibited no or slight changes throughout the berry developmental stages. These results indicated that the last three enzymatic steps of the MVA pathway might not be important control points in terpene biosynthesis.

**Figrue 5:**
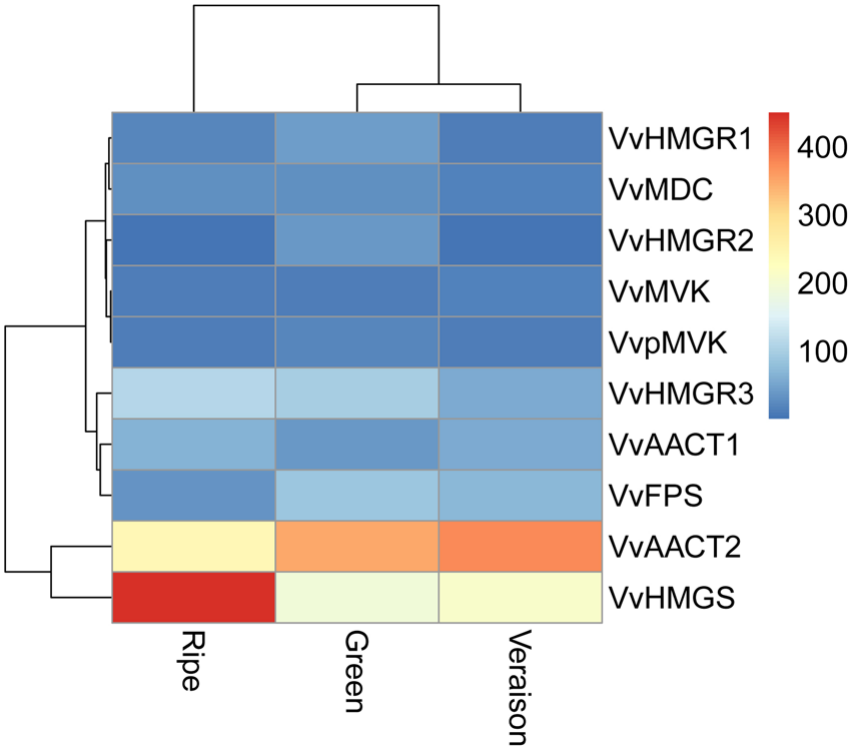
Expression level of MVA pathway genes during grapevine ripening. Data were obtained by Illumina RNA Sequencing using two biological replicas and are expressed as Reads Per Kilobase of exon model per Million mapped reads (RPKM).

### Characterization and expression analysis of genes involving in the MEP pathway

In plants, IPP and DMAPP entering in the biosynthesis of carotenoids are mainly synthesized by the MEP pathway in the plastids^12, 29, 30^. All these 13 grapevine genes were most possibly located on chloroplasts (Table 1) through protein subcellular localization prediction, which were consistent with the previous reports about the MEP pathway enzymes of *Arabidopsis* and Salvia^31, 32^.

The expressions of 11 genes involving in MEP pathway were declined during grapevine fruit development and ripening, only *VvHDR* and *VvIDI* showed an increasing expression during growth (Fig. 6 and Table S1). 1-Deoxy-D-xylulose-5-phosphate synthase (DXS) catalyzes the first committed step of the MEP pathway and carotenoid biosynthesis. Six *VvDXS* members were identified in grapevine and displayed identical expression profiles, with the lowest and highest expression levels at veraison and green stage, respectively. Interestingly, the expression level of *VvDXS1* was significantly higher than all other members (Table S1), suggesting that *VvDXS1* might play predominant role in driving grapevine fruit carotenoid accumulation. 1-deoxy-D-xylulose-5-phosphate (DXP) is converted to MEP by the enzyme DXP reductoisomerase (DXR) encoded in grapevine by single gene. The expression profile of *VvDXR* was similar to *VvDXS* with its lowest and highest expression levels at veraison and green berry stages, respectively. MEP is subsequently converted into IPP and DMAPP by the consecutive action of five independent enzymes: 4-diphosphocytidyl-2-C-methyl-D-erythritol synthase (MCT), 4-diphosphocytidyl-2-C-methyl-D-erythritol kinase (CMK), 2-C-methyl-D-erythritol 2,4-cyclodiphosphate synthase (MDS), 1-hydroxy-2-methyl-2-(E)-butenyl 4-diphosphate synthase (HDS), and 1-hydroxy-2-methyl-2-(E)-butenyl 4-diphosphate reductase (HDR)^33^. *VvMCT*, *VvCMK*, *VvMDS* and *VvHDS* showed a slight decrease in expression levels during ripening, whereas *VvHDR* demonstrated increased mRNA expression during grapevine fruit ripening (Fig. 6 and Table S1). Isomerization of IPP and DMAPP is catalyzed by isopentenyl diphosphate isomerase (IDI) encoded in grapevine by single gene whose mRNA expression profile remained strong and increased gradually throughout grapevine fruit ripening.

**Figrue 6:**
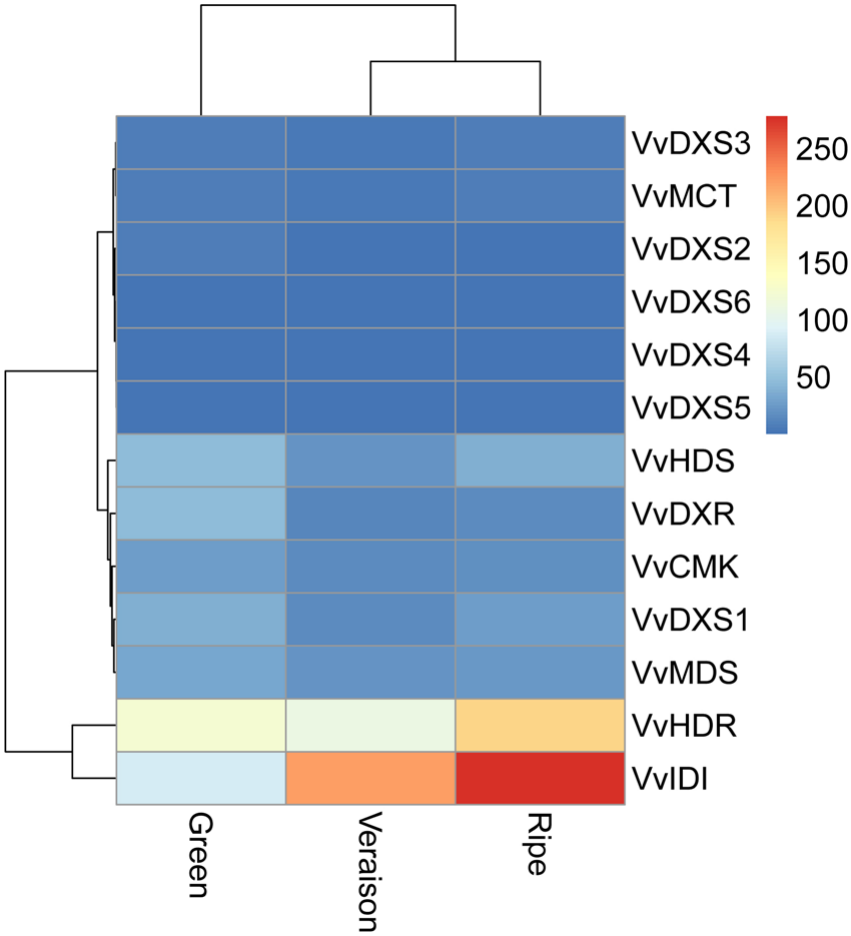
Expression level of MEP pathway genes during grapevine ripening. Data were obtained by Illumina RNA Sequencing using two biological replicas and are expressed as Reads Per Kilobase of exon model per Million mapped reads (RPKM).

### Characterization and expression analysis of carotenoid biosynthetic genes during grapevine berry development and ripening

A total of 24 genes were involved in carotenoid biosynthesis pathway, which led to the formation of carotenoids from the precursor geranylgeranyl diphosphate (GGPP). GGPP, the universal precursor for all plastid isoprenoids, is generated by geranylgeranyl diphosphate synthase (GGPPS) that catalyses the condensation of three IPP and one DMAPP units^34^. In our study, four *VvGGPPS* genes were identified and showed a steady decrease during grapevine fruit development (Fig. 7 and Table S1). *VvGGPPS2* was predominantly expressed in grapevine fruit (Fig. 7 and Table S1), in agreement with the phenomenon that *GGPPS2* was specific for chromoplasts in tomato flowers and/or fruits^35^. Phytoene synthase (PSY) the main bottleneck in the carotenoid pathway, catalyzes the condensation of two GGPP molecules to produce 15-cisphytoene^36, 37^. Three *VvPSY* genes were identified and with different exprseeion patterns in grapevine. *VvPSY1* was low at green fruit stage but started increasing at veraison, then decreased sharply at the ripening stage. In contrast, *VvPSY2* maintained low expression throughout fruit development, but increased sharply at ripening stage. *VvPSY3* was no expressed in grapevine fruit (Fig. 7 and Table S1). These results indicated that *VvPSY* genes showed a tissue-specific expression pattern.

**Figrue 7:**
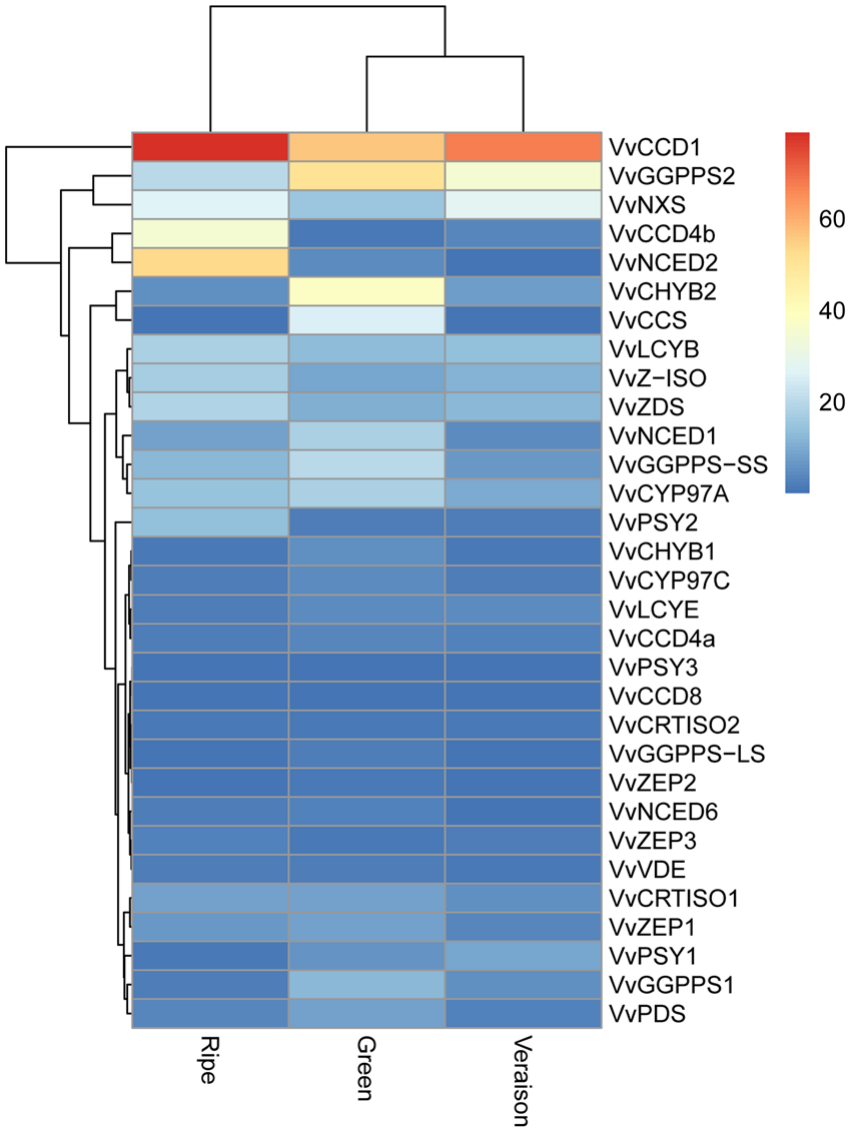
Expression level of carotenoid biosynthetic and catabolic pathway genes during grapevine ripening. Data were obtained by Illumina RNA Sequencing using two biological replicas and are expressed as Reads Per Kilobase of exon model per Million mapped reads (RPKM).

The expression level of *VvPDS* depicted highest at green fruit stage, and then showed decreasing trend from veraison till to ripe stage. The expressions of *VvZ-ISO*, *VvZDS* and *VvCRTISO* kept increasing during fruit development and reached their picks at ripening stage. Additionally, *VvCRTISO1* was much more highly expressed than *VvCRTISO2* did with the progresses in growth season (Table S1), indicating *VvCRTISO1* may have a more direct role in formation of lycopene. Furthermore, two different lycopene cyclases, lycopene β-cyclas (*VvLCYB*) and lycopene ε-cyclase (*VvLCYE*) were identified and showed an opposite expression pattern. Transcripts of *VvLCYE* decreased from veraison to harvest, whereas *VvLCYB* slightly increased through three periods of development (Table S1). The high expression levels of *VvLCYB* at harvest stage may help to maintain the amounts of γ- and β-carotene as intermediate metabolites for other compounds. Two *VvCHYB* were identified with a similar but quantitatively different expression pattern (Table S1). Neoxanthin synthase (*VvNXS*) and capsanthin-capsorubin synthase (*VvCCS*), two downstream genes of *VvCHYB*, also exhibited an opposite expression pattern. *VvNXS* were high expression at veraison and harvest stages, and *VvCCS* showed a very low expression. The expression pattern of two genes could be correlated to changes in ABA levels in grapevine berries that ABA levels typically peak at or around veraison and is thought to be responsible for the control of grape berry ripening^38, 39^.

### Characterization and expression analysis of carotenoid catabolic pathways during grapevine development and ripening

The steady-state level of carotenoids is dependent on the metabolic equilibrium between biosynthesis and degradation of carotenoids along with storage^40^. In our study, seven carotenoid cleavage dioxygenase (*VvCCD*) gene family members were identified and divided into two groups: four *VvCCDs* and three 9-cis-epoxycarotenoiddioxygenase (*VvNCEDs*). One member of *VvCCD* family (*VvCCD1*), predominantly expressed during fruit development, was found to increase and reached the highest value in the fruit ripen stage. Similar to *VvCCD1*, but to a lesser extent, *VvCCD4b* increased dramatically during grapevine development and ripening (Fig. 7 and Table S1). The strong expression of two genes may help to control carotenoid turnover and contribute to the colors or aromas of grapevine fruit. Furthermore, three *VvNCEDs* showed different expression profiles in grapevine fruit. For example, *VvNCED1* and *VvNCED6* had the highest expression at green fruit stage, while *VvNCED2* displayed the highest expression at ripe stage. The results agree with earlier studies that the expression pattern of *VvNCEDs* could not be correlated to changes in ABA levels in grapevine berries^40^. These results support the previous studies stating that different *CCDs* and *NCEDs* recognized different carotenoid substrates and cleaved at different sites, producing various apocarotenoids^41^.

### Characterization and expression analysis of chlorophyll metabolism genes during grapevine fruit development and ripening

Color change often accompanies the onset of fruit ripening and it is one of the most noticeable characteristics of grape fruit ripening. Color change in fruit typically involves chlorophyll loss and an increase in production of yellow, orange, red or purple pigments. The chlorophyll metabolic pathway can be divided into three distinct phases: (1) synthesis of chlorophyll a from glutamate; (2) interconversion between chlorophyll a and b (chlorophyll cycle); and (3) degradation of chlorophyll a into anon-fluorescent chlorophyll catabolite (Fig. S2)^42^. In this study, 29 genes involving in the three chlorophyll metabolic phases were identified (Table S2).

Twenty-one genes in chlorophyll biosynthesis, which catalyzed the formation of chlorophyll a from glutamate, exhibited gradual decrease during grapevine fruit ripening, except coproporphyrinogen III oxidase (*VvCPOX*) and protochlorophyllide reductase C (*VvPORC*) (Fig. 8 and Table S2). These results were consistent with a previous study indicating that chlorophyll degradation represented a dramatic change in metabolism at the onset of fruit ripening^43^. Further, four genes were identified in chlorophyll cycle pathway. With the exception of chlorophyll b reductase (*VvNYC1*), which was highly expressed at ripe stage, the other three genes also showed decreased expression patterns from the green fruit stage to the veraison/ripe stage. In chlorophyll degradation pathway, four genes were identified and the expression of three genes (chlorophyllase 2, *VvCLH2*; pheophytinase, *VvPPH* and pheophorbide a oxygenase, *VvPaO*) increased steadily during grapevine fruit ripening. *VvPaO*, a gene converting pheophorbide a into red chlorophyll catabolite, was highly expressed and showed a significant increase throughout the grapevine berry developmental stages, indicating that *VvPaO* may have an important role in the chlorophyll degradation. Transcriptomic datas demonstrated that the chlorophyll decline during grapevine fruit ripening was due to a acceleration in chlorophyll degradation and/or the blocking of chlorophyll synthesis.

**Figrue 8:**
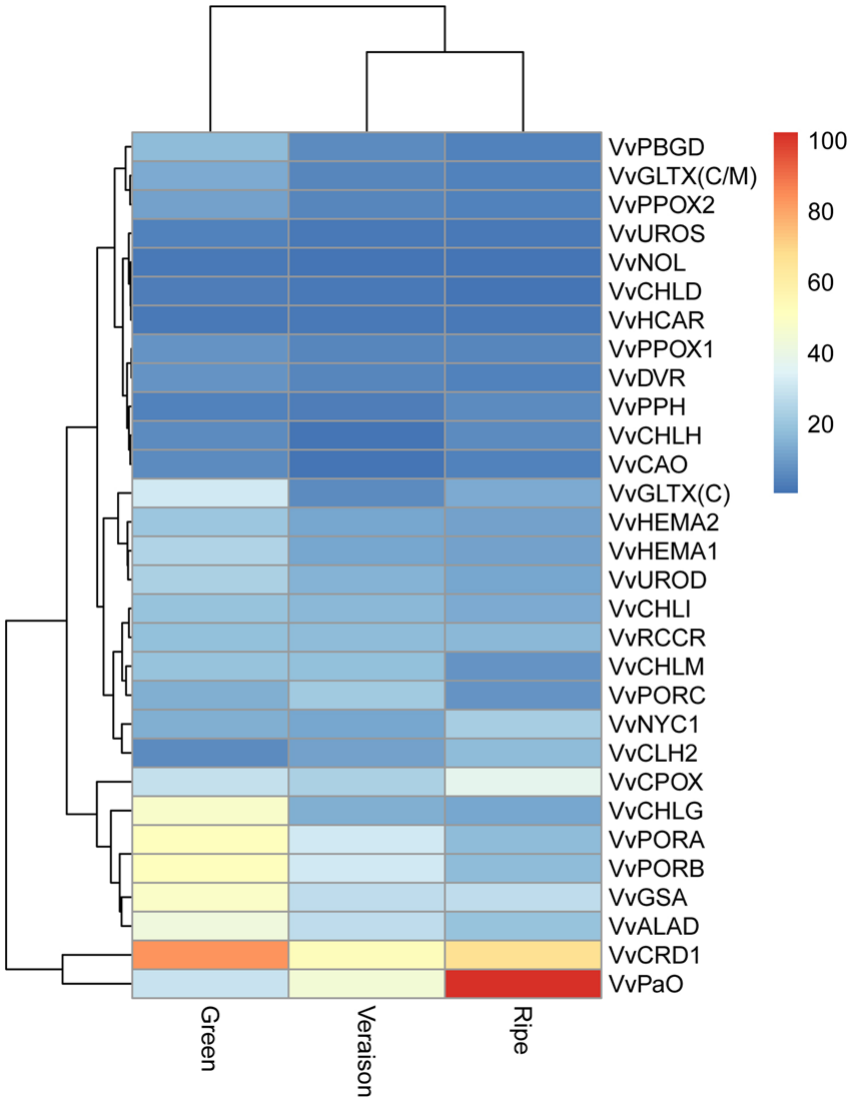
Expression level of chlorophyll metabolism genes during grapevine ripening. Data were obtained by Illumina RNA Sequencing using two biological replicas and are expressed as Reads Per Kilobase of exon model per Million mapped reads (RPKM).

### Quantitative real-time PCR validation

Eight genes involved in MVA, MEP, carotenoid biosynthesis and catabolism pathway were chosen for quantitative real-time PCR (qRT-PCR). Transcript abundance patterns were calculated over the entire course of berry development. The qRT-PCR results generally agreed (87.5%) with the changes in transcript abundance determined by RNA-seq (Fig. S3). Only *VvDXS1*, which showed lowest expression level in ripe stage, were different from that observed in RNA-seq. The qRT-PCR results suggested the reliability of the RNA-seq data.

## Discussion

Carotenoid metabolism has received much attention due to the production of the phytohormones ABA and strigolactone; as well as the formation of norisoprenoids, impact flavour and aroma compounds in a number of commercially important fruits and flowers. In grapevine, carotenoids are not only responsible for the visual appeal of grape fruit, but also enhance nutritional value and health benefits for humans^44, 45^. Thus, carotenoid content has become an important grapevine breeding objective and the study on grapevine carrotenoid metabolism has been focused. In this study, an integrative study combining carotenoid content and gene expression profiles was performed to gain insight into the synthesis and accumulation of carotenoids in grapevine during fruit development and ripening.

A toal of 54 carotenoid metabolism-related genes were identified and distributed on 17 of 19 chromosomes in grapevine, in agreement with the previous study in *Brassica rapa* ^37^ that the distribution of carotenoid metabolism genes was fractionation status at the whole-genome level. Furthermore, gene duplication and divergence events have been suggested to be the main contributors to evolutionary momentum^46^. In the current study, 11 of 54 carotenoid metabolism-related genes have arisen through either tandem or segmental duplication. Unlike *Brassica ra*pa, two pair of tandem duplication events, *VvDXS2*/*VvDXS4*/*VvDXS6* and *VvCCD4a*/*VvCCD4b*, were observed in the grapevine carotenoid genes (Fig. 2). Three pairs of segmental duplication (*VvHMGR1*/*VvHMGR3*, *VvGGPPS1*/*VvGGPPS2* and *VvCHYB1*/*VvCHYB2*) were also identified (Fig. 2). Taken together, both tandem and segmental duplications existed in grapevine carotenoid metabolism-related genes, implying that gene duplications play a major role in genomic rearrangements and expansions of grapevine carotenoid metabolism. In addition, expression differentiation was also found between duplicated genes (Table S1). For example, in three pairs of segmental duplication, one copy was much more highly expressed than the other. In other genes, *VvCCD4a* and *VvCCD4b* showed the opposite expression (Table S1). The expression differentiation between duplicate genes has been reported and it increases the probability of the retention of duplicated genes in a genome^47, 48^. These duplicated gene expression variations may be signs of subfunctionalization among different tissues and contribute to an increased complexity in the regulatory networks of the carotenoid pathway in grapevine.

Carotenoids are derived from the condensation of the 5-carbon precursors IPP and DMAPP, which are produced via the MEP pathway in plastids^49^. So far, both DXS and DXR are thought to be important rate-controlling steps of the MEP pathway and play important role in carotenoid flux regulation^6, 50^. In grapevine, six *VvDXS* genes were divided into three distinct classes (Fig. S4), suggested that each *VvDXS* gene may play different role in terpenoid biosynthesis. *VvDXS1*, the member of the DXS1 clade, was highly and predominantly expressed compared with those of other *VvDXS* genes, indicating that *VvDXS1* might play a predominant role in driving grapevine fruit carotenoid accumulation. The results are also in agreement with the previous study that Genes in the DXS1 clade played housekeeping roles and were probably involved in primary metabolism, such as the biosynthesis of carotenoids and the phytol chain of chlorophyll^51–53^. Futthermore, a positive correlation was found between the accumulation of DXR transcript and carotenoid production in *Arabidopsis* seedlings^50^ or apocarotenoids in mycorrhizal roots from monocots^54^. Similar to *Arabidopsis*, the expression of *VvDXR* was also consistent with the situation of *VvDXS1* with wide and high level expression during grapevine fruit development and ripening. In addition, the wide and high level expression of *VvDXS1* and *VvDXR* may help to maintain the amounts of IPP and DMAPP as intermediate metabolites for carotenoid compounds and contribute to the colors or aromas of grapevine fruit.

The cytoplasmic MVA pathway also contribute to the synthesis of IPP and DMAPP (Fig. S1), which provide precursors for the biosynthesis of sesquiterpenes, polyterpenes, sterols, dolichol, and for ubiquinone formation in mitochondria. In our sthdy, the expression of *VvAACT2* was significantly higher than that of *VvAACT1* (5.8-fold), suggesting that *VvAACT2* may play a more important role in cytosolic isoprenoid biosynthesis during grapevine berry development and ripening. Similar to grapevine, Arabidopsis *AACT2* (*AtAACT2*) was six times more efficient than *AtAACT1* did in complement the *erg10* yeast mutant lacking *AACT* ^55^. Three *VvHMGRs*, the rate-limiting step in MVA pathway, showed a completely differential expression pattern throughout the berry developmental stages, in agreement with previous studies that differential expressions of HMGR isozymes had a critical regulatory role for channeling and counter balancing carbon flux to the different downstream pathways of development or stress response^56^.

GGPPS is an important branch point enzyme in terpenoid biosynthesis since their enzymatic product GGPP is the direct precursor not only carotenoids, but also diterpenes, ABA and other compounds^57^. In grapevine, four divergent *VvGGPPS* isoforms showed different subcellular localization consistent with the subcellular compartmentalization of the diverse GGPP-dependent terpenoid pathways. *VvGGPPS2* showed significantly higher expression level than any other member, indicating that *VvGGPS2* had essential functions in carotenoid biosynthetic pathway during grapevine fruit development. A decreasing expression of *VvGGPS2* from green to the red ripe stage had a significant positive correlation with a decrease of the total carotenoid content. PSY is a rate-limiting enzyme of carotenoid biosynthesis and is frequently regarded as the major bottleneck in carbon flux to carotenoids^15^. For example, over-expressing tomato *PSY1* (*SlPSY1*) significantly increased the carotenoid content in tomato fruit^58^. In this study, three *VvPSY* exhibited different expression pattern. Unlike *SlPSY1* with most highly expressed in ripe tomato fruits^59^, *VvPSY1* was highly expressed in green and veraison stages, whereas *VvPSY2* showed the highest expression in ripe grapevine fruits, confirming the complex regulation of the pathway. *VvPSY3*, a homologly with tomato *SlPSY3* (Fig. S5), was not expressed in fruit, suggesting that *VvPSY3* should be associated with root carotenogenesis, because *SlPSY3* mainly expressed in root^60^. Furthermore, the cyclization of lycopene is a central branch point in the carotenoid biosynthetic pathway (Fig. S1). The expression of *VvLCYB* gradually increased and was significantly higher than that of *VvLCYE* during grapevine fruit ripening, especially in ripe stage of *VvLCYB* (Table S1). The induction of *VvLCYB* during grapevine fruit development and ripening might be responsible for maintain the amounts of β-carotene as intermediate metabolites for other compounds, such as ABA and C13-norisoprenoids. In grapevine, ABA concentrations increase and peak at the onset of ripening^61^. The gene expression profiles of *VvNXS* and *VvCCS* seem to support the phenomenon. The low expression of *VvCCS* at veraison and harvest stages created a blockade downstream, and the high expression of *VvNXS* might help to accumulate the neoxanthin, which is an intermediate in the biosynthesis of the plant hormone ABA.

The steady-state levels of carotenoid is dependent on the balance between biosynthesis and degradation along with storage^15^. Consistent with Arabidopsis *CCD* ^62^, seven *VvCCD* family genes were divided into two groups, including four *VvCCDs* and three *VvNCEDs* (Table S1). *VvCCD1* and *VvCCD4b* were found to increase stably and reached the highest values in the ripe stage. The significantly negative correlation between carotenoid content and gene expression profiles indicated that *VvCCD1* and *VvCCD4b* preferentially involved in volatile compound production from different carotenoid substrates during grapevine fruit development and ripening. This negative correlation between the expression of *CCD1* and *CCD4* and carotenoid levels also exists in chrysanthemum flowers, potato, and strawberry^63–65^. Taken together, these results suggested that expression profile of genes coding for carotenoid biosynthetic pathway associated with the accumulation of carotenoid in grapevine fruit.

## Conclusions

Fifty-four carotenoid biosynthetic genes were identified in grapevine genome. The composition of carotenoid biosynthetic genes could explain the metabolic profiles of carotenoid accumulation and help to elaborate the genetic mechanism of carotenoid biosynthesis in grapevine. The expression analysis of carotenoid biosynthetic genes showed that down regulation of *VvDXS*, *VvDXR*, *VvGGPPS* and *VvPSY* and a dramatic increase in the transcription levels of *VvCCD* were responsible for the reduction of carotenoids. The visible correlation between carotenoid content and gene expression profiles also suggested that transcriptional regulation of carotenoid biosynthesis pathway genes is a key mechanism of carotenoid accumulation. These results represent an important reference study for further characterisation of carotenoid biogenesis and accumulation in grapevine.

## Materials and Methods

### Plant material

Three-years-old ‘Fujiminori’ grapevine trees, grown in the standard field conditions at the Nanjing Agricultural University fruit farm, Nanjing, China, were chosen as the experimental material. Berries samples were collected at three time points: green stage (40 DAF), veraison (65 DAF) and ripe/harvest stages (90 DAF) throughout the growing season. Furthermore, in order to capture a representative biological selection of transcripts at each time-point, RNA for Illumina sequencing was purified from tissues of 40 berries sampled from 20 bunches.

### Identification and analysis of the carotenoid biosynthesis-related genes in grapevine

Carotenoid biosynthesis-related genes were identified via BLAST-P search in NCBI using the Arabidopsis gene sequences as queries. Gene sequences of Arabidopsis involved in the carotenoid biosynthetic pathway were acquired from the TAIR database (www.arabidopsis.org) and KEGG pathway database (http://www.genome.jp/kegg/pathway.html). The grapevine genome and a set of annotated gene sequences from Grape genome database (http://genomes.cribi.unipd.it/grape/) were used to identify the carotenoid biosynthetic genes in grapevine. Subsequently, to verify the reliability of the initial results, a survey was conducted to confirm these genes using functional annotation of the grapevine transcriptome in three distinct stages of berry development (NCBI GEO Accession: GSE77218)^19^.

### Determination of carotenoid and chlorophyll content

Accurately weighted 0.5 g of fresh grapevine sample was taken, and homogenized in tissue homogenizer with 10 ml of 95% ethanol. Homoginized sample mixture was centrifuge for 10,000 rpm for 15 min at 4 °C. The supernatant were separated and 0.5 ml of it is mixed with 4.5 ml of the 95% ethanol. The solution mixture was analyzed for Chlorophyll a, Chlorophyll b and carotenoids content in spectrophotometer (Parkin). The chlorophyll a, Chlorophyll b and carotenoid are respectively determined by spectrophotometric measurement at 664, 649and 470 nm as previous research^66^.

### Sequence feature analysis of grapevine carotenoid biosynthesis-related genes

The genomic sequence for each carotenoid metabolism gene was extracted from the whole genomic sequence according to gene location in the chromosome in the annotation file using a programmed Perlscript. Intron/exon structures were predicted using the Gene Structure Display Server (http://gsds.cbi.pku.edu.cn/chinese.php). The molecular weight and theoretical isoelectric point were predicted using the Prot-Param analyses (http://cn.expasy.org/tools/protparam.html)^67^ on the basis of their sequence. Conserved domains were searched using the Conserved Domain Database (http://www. ncbi.nlm.nih.gov/Structure/cdd/wrpsb.cgi). The localizations of deduced proteins were predicted on the TargetP1.1 server (http://www.cbs.dtu.dk/services/TargetP/)^68^.

### Chromosomal locations, gene duplication and phylogenetic analysis of grapevine carotenoid biosynthesis-related genes

The information of chromosomal position of each carotenoid biosynthesis-related genes was obtained from the grapevine genome (http://www.genoscope.cns.fr/externe/GenomeBrowser/Vitis/), and then the map was drafted using MapInspect software (http://www.plantbreeding.wur.nl/uk/software-mapinspect.html). MCScanX software (http://chibba.pgml.uga.edu/mcscan2/) was used to detect the gene duplication events, with the E-value set below 1 × 10^−5^ following the description in a previous study^69^. The diagrams were generated by the program Circos version 0.63 (http://circos.ca/). Phylogenetic trees were constructed in MEGA7.0 software using the neighbor-joining (NJ) method and maximum likelihood (ML) method with 1000 bootstrap replications.

### Expression analysis of grapevine carotenoid biosynthesis-related genes

The expression patterns of carotenoid biosynthetic genes in grapevine were acquired from gene expression omnibus (GEO) database of NCBI (GSE77218), which measured using RNA-Seq data^19^. Differential expression was analyzed and calculated based on the count values of each transcript between libraries using edgeR (the Empirical analysis of Digital Gene Expression in R) software^70^. The thresholds for judging significant differences in transcript expression were “FDR < 0.001” and “|log2 fold-change (log2FC)| ≥ 1”. Transcripts with |log_2_FC| < 0.25 were assumed to no change in expression levels. Other transcripts (0.25 < |log2FC| < 1) were considered as “up-regulated slightly” or “down-regulated slightly”.

### qRT-PCR validation

qRT-PCR was performed to verify the expression patterns revealed by the RNA-seq study. Total RNA samples of three stages of grapevine fruit development were extracted using Trizol reagent (Invitrogen, Carlsbad, CA, USA). Purified RNA samples were reverse-transcribed using the PrimeScript RT Reagent Kit with gDNA Eraser (Takara, Dalian, China) as per the manufacturer’s protocol. Eight transcripts were selected for the qRT-PCR assay. Gene specific qRT-PCR primers were designed using Primer3 software (http://primer3.ut.ee/) (Table S3). qRT-PCR was carried out using an ABI PRISM 7500 real-time PCR system (Applied Biosystems, USA). Each reaction mix was composed of 10 μl 2 × SYBR Green Master Mix Reagent (Applied Biosystems, USA), 2.0 μl cDNA sample, and 400 nM of gene-specific primers in a final volume of 20 μl. PCR conditions were: 2 min at 95 °C, followed by 40 cycles of denaturation at 95 °C for 10 s and annealing at 60 °C for 40 s. The relative mRNA level for each gene was calculated using the 2^−ΔΔCT^ formula^71^. A primer pair was also designed for TC81781 (The Institute for Genomic Research, Release 6.0), encoding an actin protein (housekeeping gene). At least three replicates of each cDNA sample were performed for qRT-PCR analysis.

## Electronic supplementary material

Supplementary information

Table S1

Table S2

Table S3
