# Supplementary material for: Genome-wide identification and characterization of genes involved in carotenoid metabolic in three stages of grapevine fruit development
Source: Sci Rep. 2017 Jun 26;7:4216. doi: 10.1038/s41598-017-04004-0 (PMC5484692; doi:10.1038/s41598-017-04004-0)
Supplement: Supplementary file 1 — Supplementary information [file 41598_2017_4004_MOESM1_ESM.pdf]

Manuscript title :

Genome-wide identification and characterization of genes involved in carotenoid metabolism in three stages of grapevine fruit development

Author list:

Xiangpeng Leng, Peipei Wang, Chen Wang, Xudong Zhu, Xiaopeng Li, Hongyan Li, Qian Mu, Ao Li, Zhongjie Liu & Jinggui Fang

Supplementary tables:

Table S1 The gene expression patterns of carotenoid metabolic pathway genes in three stages of grapevine fruit development.

Table S2 The gene expression patterns of chlorophyll synthesis and degradation pathway genes in three stages of grapevine fruit development.

Table S3 The primers sequences of carotenoid biosynthesis genes for qRT-PCR.

Supplementary figures:

Fig. S1 Summary diagram of carotenoid metabolism pathways in grapevine (MVA, MEP, carotenoid biosynthetic and catabolic pathways). MVA pathway: *ACAT*, acetyl-CoA acetyltransferase; *HMGS*, 3-hydroxy-3-methylglutaryl-CoA synthase; *HMGR*, 3-hydroxy-3-methylglutaryl-CoA reductase; *MVK*, mevalonate kinase; *pMVK*, phosphomevalonate kinase; *MDC*, mevalonate diphosphate decarboxylase; *FPS*, farnesyl diphosphate synthase; MEP pathway: *DXS*, 1-deoxy-D-xylulose-5-phosphate synthase; *DXR*, 1-deoxy-D-xylulose-5-phosphate reductoisomerase; *MCT*, 4-diphosphocytidyl-2-C-methyl-D-erythritol synthase; *CMK*, 4-diphosphocytidyl-2-C-methyl-D-erythritol kinase; *MDS*, 2-C-methyl-D-erythritol 2,4-cyclodiphosphate

synthase; *HDS*, 1-hydroxy-2-methyl-2-(E)-butenyl 4-diphosphate synthase; *HDR*, 1-hydroxy-2-methyl-2-(E)-butenyl 4-diphosphate reductase; *IDI*, isopentenyl diphosphate isomerase; carotenoid biosynthetic pathway: *GGPPS*, geranylgeranyldiphosphate synthase; PSY, phytoene synthase; PDS, phytoene desaturase; *Z-ISO*, ζ-carotene isomerase; *ZDS*, ζ-carotene desaturase; *CRTISO*, carotene isomerase; *LCYE*, lycopene ε-cyclase; *LCYB*, lycopene β-cyclase; *CHYB*, β-carotene hydroxylase; *CYP97C*, cytochrome P450-type monooxygenase 97C; *CYP97A*, cytochrome P450-type monooxygenase 97A (LUTEIN DEFICIENT 5); *ZEP*, zeaxanthin epoxidase; *VDE*, violaxanthin de-epoxidase; *NXS*, neoxanthin synthase; Carotenoid catabolic, *NCED*, 9-cis-epoxycarotenoiddioxygenase; *CCS*, capsanthin-capsorubin synthase; *CCD*, carotenoid cleavage dioxygenase. The asterisks identify enzymes encoded by several gene isoforms.

Fig. S2 Summary diagram of chlorophyll metabolic pathways in grapevine. Genes encode the following enzymes. Chlorophyll synthesis: *GLTX*, glutamyl-tRNA synthetase; *HEMA*, glutamyl-tRNA reductase; *GSA*, glutamate-1-semialdehyde 2,1-aminomutase; *ALAD*, Delta-aminolevulinic acid dehydratase; *PBGD*, porphobilinogen deaminase; *UROS*, uroporphyrinogen-III synthase; *UROD*, uroporphyrinogen decarboxylase; *CPOX*, coproporphyrinogen III oxidase; *PPOX*, protoporphyrinogen oxidase; *CHLH/G/I*, magnesium-chelatase H/D/I subunit; *CHLM*, magnesium protoporphyrin IX methyltransferase; *CRD1*, magnesium-protoporphyrin IX monomethylester [oxidative] cyclase; *PORA/PORC*, protochlorophyllide reductase A/C; *DVR*, divinyl chlorophyllide a 8-vinyl-reductase; *CHLG*, chlorophyll synthase; Chlorophyll cycle: *CAO*, chlorophyllide a oxygenase; *NYC1*, chlorophyll b reductase; *NOL*, chlorophyll b reductase-like; *HCAR*, 7-hydroxymethyl chlorophyll a reductase; Chlorophyll degradation: *CLH2*, chlorophyllase 2; *PPH*, pheophytinase; *PaO*,

pheophorbide a oxygenase; *RCCR*, red chlorophyll catabolite reductase. *MCS*, metal-chelating substance. In the diagram, the enzymes with down-regulated gene expression level are indicated in black, the enzymes with up-regulated gene expression level in red, while the enzymes with no change in blue. The asterisks identify enzymes encoded byseveral gene isoforms.

Fig. S3 qRT-PCR validation of differentially expressed transcripts from RNA-seq.

Fig. S4 Phylogenetic relationship of plant DXSs. The rooted Neighbor-Joining tree was constructed using the MEGA program (version 7.0) with default parameters. DXSs included are Vitis vinifera *Vv*DXS1 (VIT_05s0020g02130.t01), *VvDXS2* (VIT_11s0052g01730.t01), *VvDXS3* (VIT_04s0008g04970.t01), *VvDXS4* (VIT_11s0052g01780.t01), *VvDXS5* (VIT_00s0218g00110.t0), *VvDXS6* (VIT_11s0052g01240.t01); Arabidopsis thaliana *AtDXS1* (At4g15560), *AtDXS2* (At3g21500), *AtDXS3* (At5g11380); Oryza sativa *OsDXS1* (NP_001055524), *OsDXS2* (NP_001059086), *OsDXS3* (BAA83576); Populus trichocarpa *PtDXS1* (XP_002312717), *PtDXS2A* (XP_002303416), *PtDXS2B* (XP_002331678), *PtDXS3* (XP_002308644); Salvia miltiorrhiza *SmDXS1* (EU670744), *SmDXS2* (FJ643618), *SmDXS3* (JN831116), *SmDXS4* (JN831117), *SmDXS5* (JN831118).

Fig. S5 Phylogenetic relationships of PSYs form Grapevine and various other plants. PSYs included are Vitis vinifera *VvPSY1* (VIT_04s0079g00680.t01), *VvPSY2* (VIT_12s0028g00960.t01), *VvPSY3* (VIT_06s0004g00820.t01); Arabidopsis thaliana *AtPSY* (AT5G17230); Solanum lycopersicum *SlPSY1* (Solyc03g031860), *SlPSY2* (Solyc02g081330), *SlPSY3* (Solyc01g005940); Oryza sativa *OsPSY1* (AAS18307), *OsPSY2* (AAK07735), *OsPSY3* (DQ356431); Zea mays *ZmPSY1* (P49085), *ZmPSY2* (AAQ91837), *ZmPSY3* (DQ356430).

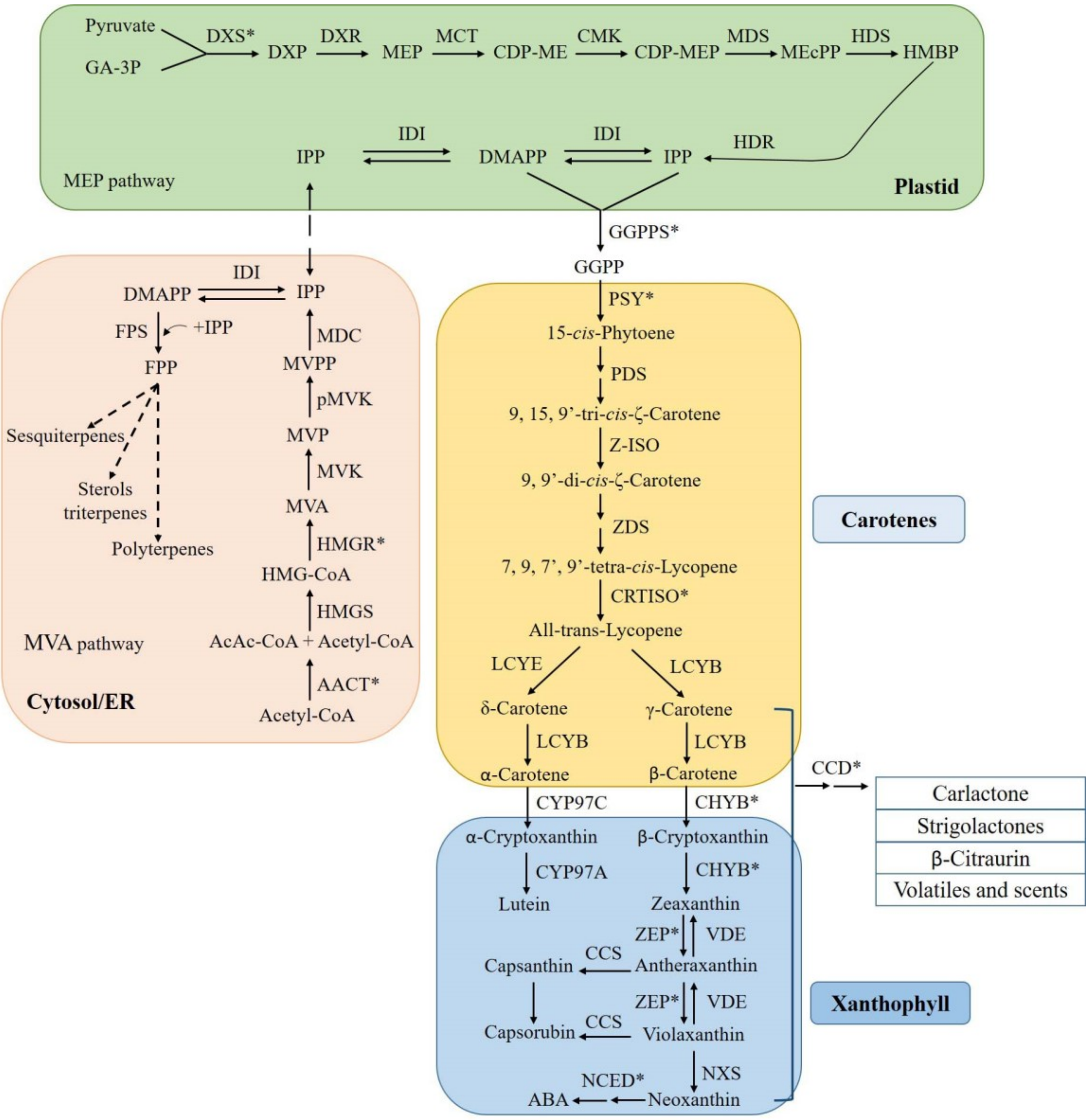
Pyruvate
GA-3P
DXS*
DXP
DXR
MEP
MCT
CDP-ME
CMK
CDP-MEP
MDS
MEcPP
HDS
HMBP
IPP
IDI
DMAPP
IDI
IPP
HDR
MEP pathway
Plastid
GGPPS*
GGPP
IDI
DMAPP
IPP
FPS
+IPP
MDC
FPP
MVPP
pMVK
Sesquiterpenes
MVP
Sterols
triterpenes
MVK
MVA
HMGR*
Polyterpenes
HMG-CoA
HMGS
MVA pathway
AcAc-CoA + Acetyl-CoA
AACT*
Cytosol/ER
Acetyl-CoA
PSY*
15-cis-Phytoene
PDS
9, 15, 9'-tri-cis-ζ-Carotene
Z-ISO
9, 9'-di-cis-ζ-Carotene
ZDS
Carotenes
7, 9, 7', 9'-tetra-cis-Lycopene
CRTISO*
All-trans-Lycopene
LCYE
LCYB
δ-Carotene
γ-Carotene
LCYB
LCYB
α-Carotene
β-Carotene
CCD*
Carlactone
Strigolactones
β-Citraurin
Volatiles and scents
CYP97C
CHYB*
α-Cryptoxanthin
β-Cryptoxanthin
CYP97A
CHYB*
Lutein
Zeaxanthin
ZEP*
VDE
CCS
Capsanthin
Antheraxanthin
ZEP*
VDE
Xanthophyll
CCS
Capsorubin
Violaxanthin
NXS
NCED*
ABA
Neoxanthin

Fig. S1

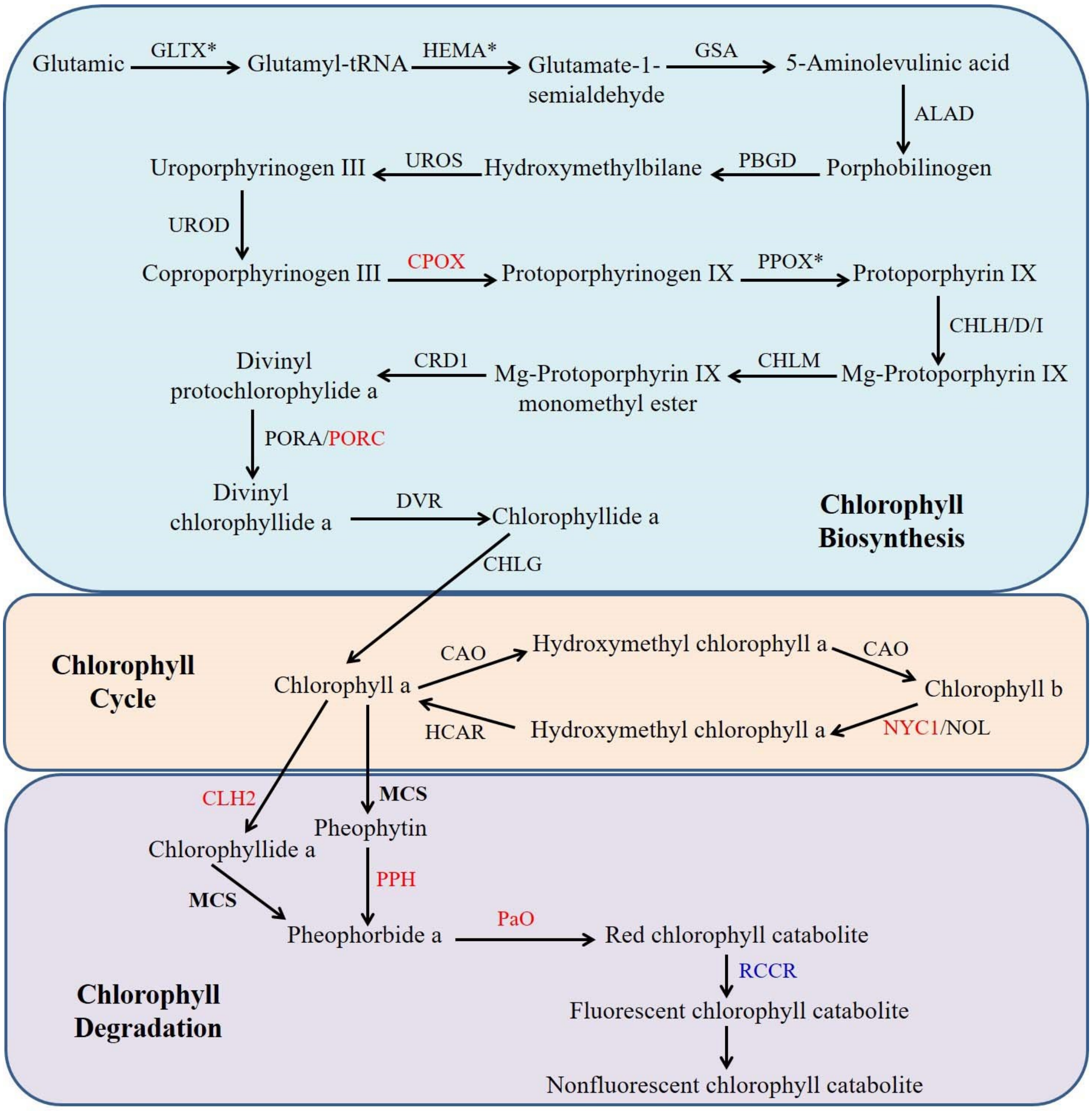
Glutamic
GLTX*
Glutamyl-tRNA
HEMA*
Glutamate-1-semialdehyde
GSA
5-Aminolevulinic acid
ALAD
Porphobilinogen
PBGD
Hydroxymethylbilane
UROS
Uroporphyrinogen III
UROD
Coproporphyrinogen III
CPOX
Protoporphyrinogen IX
PPOX*
Protoporphyrin IX
CHLH/D/I
Mg-Protoporphyrin IX
CHLM
Mg-Protoporphyrin IX monomethyl ester
CRD1
Divinyl protochlorophylide a
PORA/PORC
Divinyl chlorophyllide a
DVR
Chlorophyllide a
Chlorophyll Biosynthesis
CHLG
Chlorophyll Cycle
Chlorophyll a
CAO
Hydroxymethyl chlorophyll a
CAO
Chlorophyll b
NYC1/NOL
Hydroxymethyl chlorophyll a
HCAR
CLH2
MCS
Chlorophyllide a
Pheophytin
MCS
PPH
Pheophorbide a
PaO
Red chlorophyll catabolite
RCCR
Fluorescent chlorophyll catabolite
Nonfluorescent chlorophyll catabolite
Chlorophyll Degradation

Fig. S2

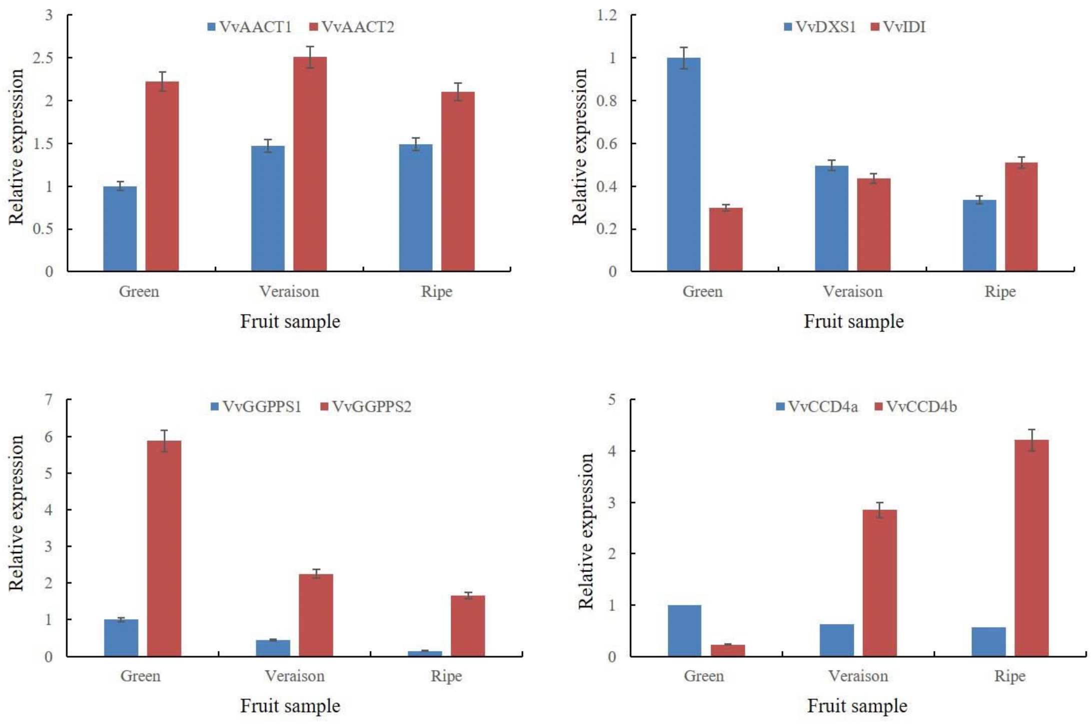
VvAACT1
VvAACT2
Relative expression
Green
Veraison
Ripe
Fruit sample
VvDXS1
VvIDI
Relative expression
Green
Veraison
Ripe
Fruit sample
VvGGPPS1
VvGGPPS2
Relative expression
Green
Veraison
Ripe
Fruit sample
VvCCD4a
VvCCD4b
Relative expression
Green
Veraison
Ripe
Fruit sample

Fig. S3

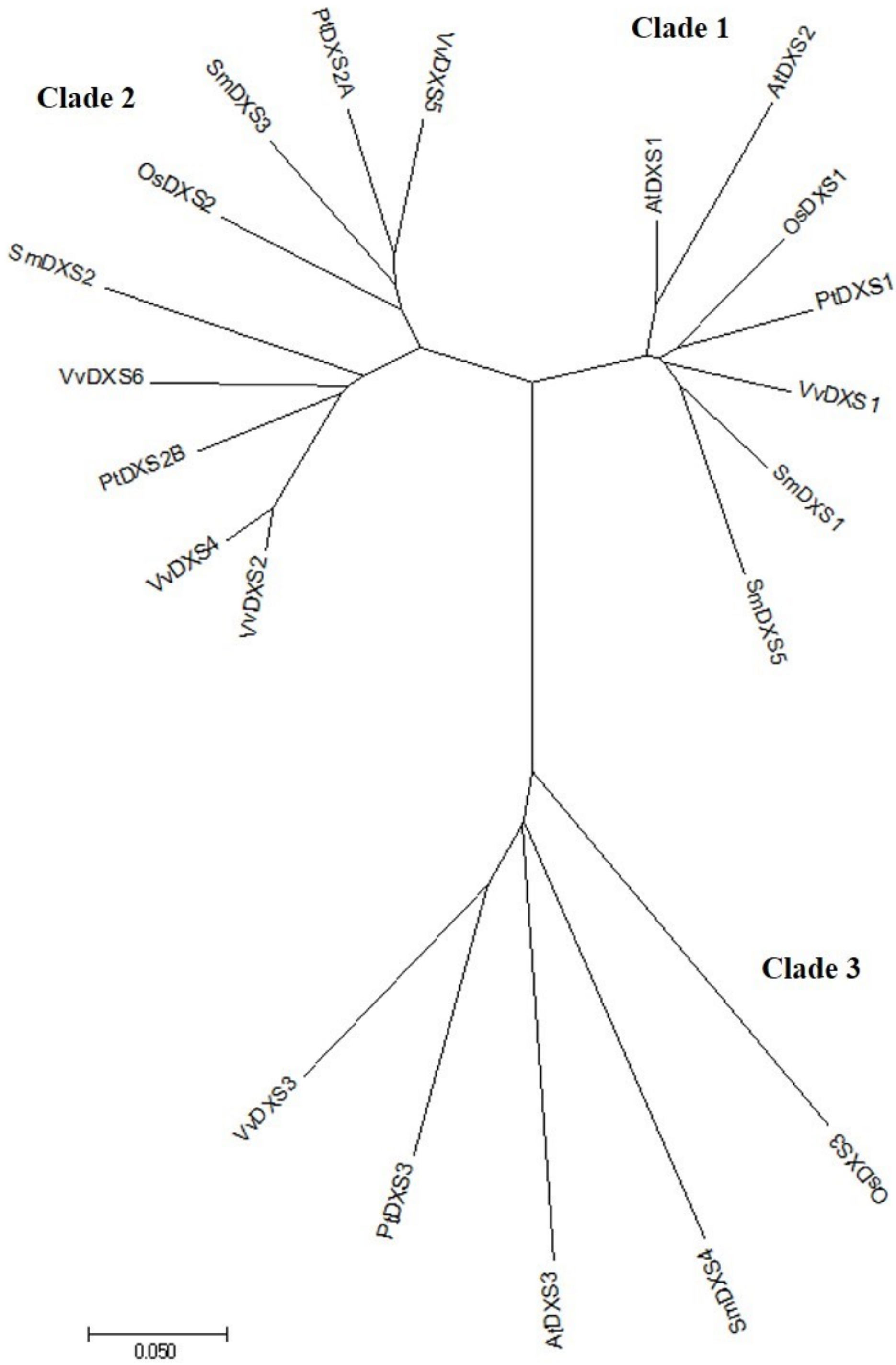
Clade 1
Clade 2
Clade 3
AtDXS2
AtDXS1
OsDXS1
PtDXS1
VvDXS1
SmDXS1
SmDXS5
PtDXS2A
VvDXS5
SmDXS3
OsDXS2
SmDXS2
VvDXS6
PtDXS2B
VvDXS4
VvDXS2
VvDXS3
PtDXS3
AtDXS3
SmDXS4
OsDXS3
0.050

Fig. 4

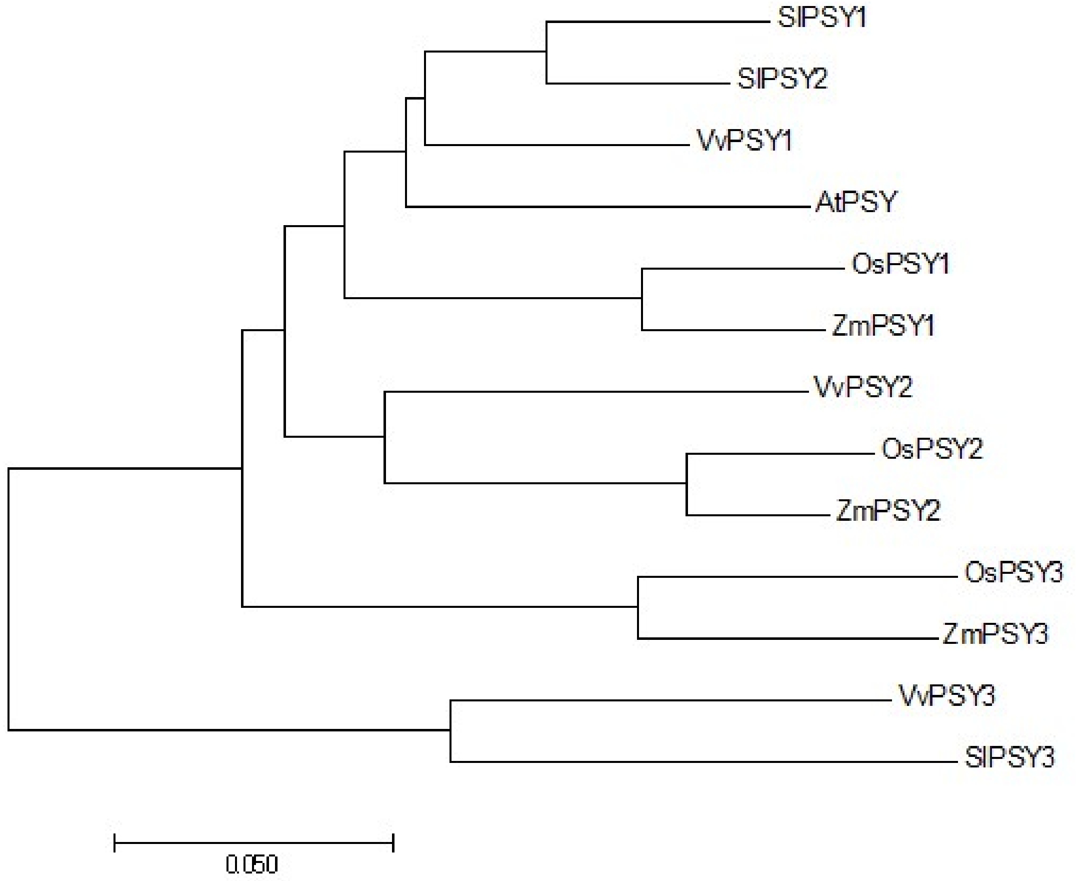
SlPSY1
SlPSY2
VvPSY1
AtPSY
OsPSY1
ZmPSY1
VvPSY2
OsPSY2
ZmPSY2
OsPSY3
ZmPSY3
VvPSY3
SlPSY3
0.050

Fig. S5
