# Supplementary material for: Genome-wide identification and characterization of genes involved in carotenoid metabolic in three stages of grapevine fruit development
Source: Sci Rep. 2017 Jun 26;7:4216. doi: 10.1038/s41598-017-04004-0 (PMC5484692; doi:10.1038/s41598-017-04004-0)
Supplement: Supplementary file 4 — Table S3 [file 41598_2017_4004_MOESM4_ESM.doc]

**Table S3. The primers sequences of carotenoid biosynthesis genes for qRT-PCR**

| Gene name | Gene ID | Forward primer sequences (5'-3') | Reverse primer sequences (5'-3') |
| --- | --- | --- | --- |
| VvAACT1 | VIT_00s0531g00050.t01 | GGATCTCGATTAGGGCATGA | GTGCAGAAATACCACGCTCA |
| VvAACT2 | VIT_12s0057g01200.t01 | GTGATGGTGCTGCTGCTTTA | CTGATTTGCAAGAGCCACAA |
| VvDXS1 | VIT_05s0020g02130.t01 | CCAGGTGGTGCATGATGTAG | AAACAACTGGGCCTGTCATC |
| VvIDI | VIT_04s0023g00600.t01 | TGCTGCACAGAGCTTTCAGT | TCAACAGGTGCATCTTCAGC |
| VvGGPPS1 | VIT_04s0023g01210.t01 | TCCGTCCCGTTCTCTGTATC | CCTTGTGATTTGTGGGCTTT |
| VvGGPPS2 | VIT_18s0001g12000.t01 | GGAACAAAGACGCCACATTT | AGCCTCGTCCAAGGCTTTAT |
| VvCCD4a | VIT_02s0087g00910.t01 | CCACACGGAATCTGGACTTT | TGGATTATCTGGCTCCCTTG |
| VvCCD4b | VIT_02s0087g00930.t01 | ATCTGAAAACGGGGACAGTG | CCTTTGCCACAAAGAATGGT |
